# Supplementary material for: Obesity-Related Genes Expression in Testes and Sperm Parameters Respond to GLP-1 and Caloric Restriction
Source: Biomedicines. 2022 Oct 17;10(10):2609. doi: 10.3390/biomedicines10102609 (PMC9599882; doi:10.3390/biomedicines10102609)
Supplement: Supplementary file 1 [file biomedicines-10-02609-s001.zip › biomedicines-1929224-supplementary.pdf]

Table S1. ORGs abundance association with sperm and testis oxidative status, other ORGs abundance, sperm-quality parameters, and HOMAR-IR, in rats under CR. All correlations were assessed by computing Pearson correlation coefficients (r) assuming Gaussian distribution (confidence interval of 95%). All the correlations represented have a p value < 0.05.

		ORGs abundance (relative expression)					
		Testis *FTO*	Sperm *FTO*	Sperm *MC4R*	Testis *GNPDA2*	Testis *TMEM18*	Sperm *TMEM18*
Oxidative Status	**Testis *NFE2L2***	$p = 0.0170$ $r = 0.8916$		$p = 0.0082$ $r = 0.9251$		$p = 0.0185$ $r = 0.8868$	
	**Sperm *NFE2L2***		$p = 0.0113$ $r = 0.9121$				$p = 0.0001$ $r = 0.9904$
	**Sperm TAC**	$p = 0.0218$ $r = 0.8769$		$p = 0.0462$ $r = 0.8189$		$p = 0.0412$ $r = 0.8293$	
ORGs abundance (relative expression)	**Testis *MC4R***	$p = 0.0125$ $r = 0.9521$					
	**Sperm *MC4R***	$p = 0.0008$ $r = 0.9761$					
	**Testis *TMEM18***	$p = 0.020$ $r = 0.9630$					
	**Sperm *TMEM18***		$p = 0.0031$ $r = 0.9539$				
	**HOMAR-IR**					$p = 0.0044$ $r = -0.9452$	
Sperm quality parameters	**Sperm head defects**				$p = 0.0393$ $r = 0.8334$		
	**Sperm Concentration**		$p = 0.0015$ $r = 0.9685$				$p = 0.0006$ $r = 0.9804$
	**Sperm Viability**					$p = 0.0258$ $r = 0.8657$	

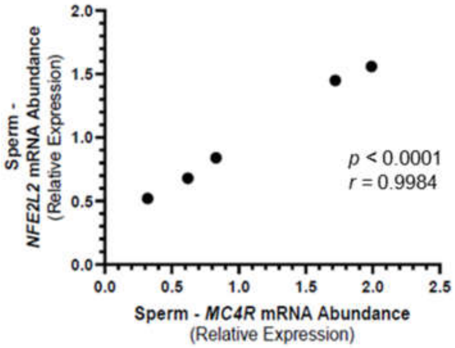

Figure S1. *MC4R* abundance indirect association with the sperm oxidative status in rats subjected to GLP- 1 administration. Association of *MC4R* mRNA abundance and the *NFE2L2* mRNA abundance in sperm of rats subjected to GLP-1 administration was evaluated by computing Pearson correlation coefficients (r) assuming Gaussian distribution (confidence interval of 95%). All p values < 0.05 were considered statistically significant.

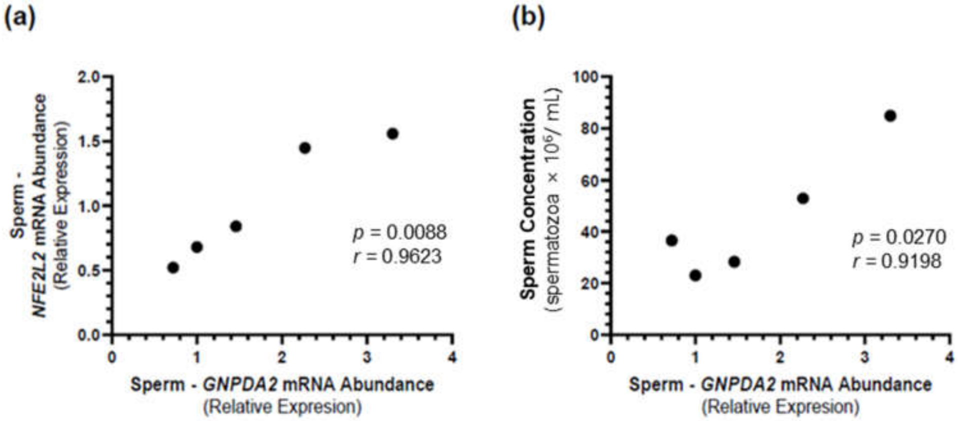

Figure S2. *GNPDA2* abundance association with the sperm oxidative status and concentration in the rats subjected to GLP-1 administration. Associations between *NFE2L2* mRNA abundance in sperm (**a**) and sperm concentration (**b**) with *GNPDA2* mRNA abundance also in sperm. The associations were evaluated by computing Pearson correlation coefficients (*r*) assuming Gaussian distribution (confidence interval of 95%). All P values < 0.05 were considered statistically significant.

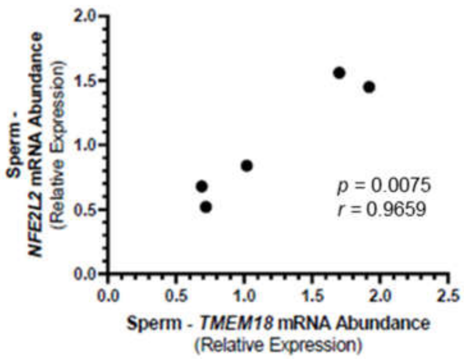

Figure S3. *TMEM18* abundance indirect association with the sperm oxidative status in rats subjected to GLP-1 administration. Association between *TMEM18* mRNA abundance and the *NFE2L2* mRNA abundance in sperm was evaluated by computing Pearson correlation coefficients (*r*) assuming Gaussian distribution (confidence interval of 95%). All *p* values < 0.05 were considered statistically significant.

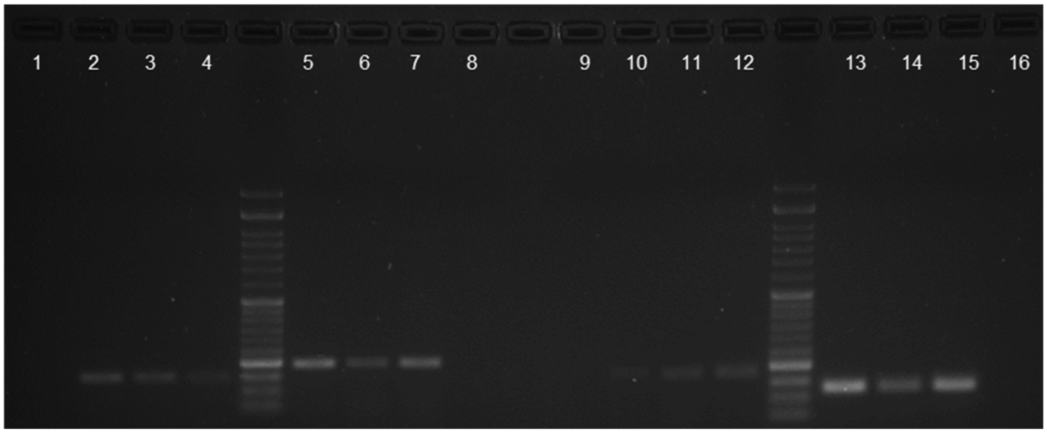

Figure S4. Original PCR gel image from the identification of ORG-associated proteins in rat testis and spermatozoa. *FTO* (151 bp) (1) negative control for *FTO*; (2) *FTO* present in rat sperm; (3) *FTO* present in rat testis; (4) *FTO* present in rat brain; *MC4R* (192 bp) (5) *MC4R* present in rat brain; (6) *MC4R* present in rat testis; (7) *MC4R* present in rat sperm; (8) negative control for *MC4R; GNPDA2* (181 bp) (9) negative control for *GNPDA2*; (10) *GNPDA2* present in rat sperm; (11) *GNPDA2* present in rat testis; (12) *GNPDA2* present in rat brain; *TMEM18* (127 bp) (13) *TMEM18* present in rat brain; (14) *TMEM18* present in rat testis; (15) *TMEM18* present in rat sperm; (16) negative control for *TMEM18*.

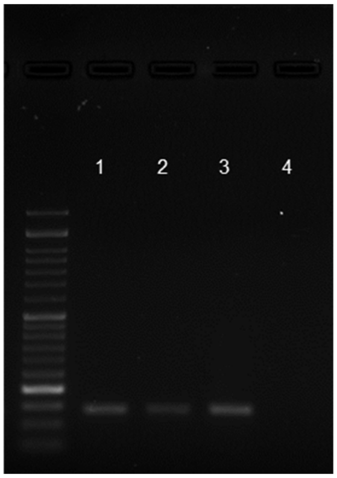

Figure S5. Original PCR gel image from the identification of NFE2L2 in rat testis and spermatozoa. *NFE2L2* (139 bp) (1) *NFE2L2* present in rat brain; (2) *NFE2L2* present in rat testis; (3) *NFE2L2* present in rat sperm; (4) negative control for *NFE2L2*.
